# Associations of Anthropometric Indicators with Bone Mineral Density and the Risk of Bone Fractures and Falls: A Large-Scale Genetic Correlation Study

**DOI:** 10.2174/0118715303369995250702111723

**Published:** 2025-07-18

**Authors:** Xiao Hu, Zhao-Xing Gao, Yan-Yu Zhu, Sheng Li, Wen-Wen Ding, Hai-Feng Pan, Peng Wang

**Affiliations:** 1 Department of Health Promotion and Behavioral Sciences, School of Public Health, Anhui Medical University, 81 Meishan Road, Hefei 230032, Anhui, China;; 2 Institute of Kidney Disease, Inflammation & Immunity Mediated Diseases, the Second Hospital of Anhui Medical University, Hefei 230032, Anhui, China;; 3 Department of Epidemiology and Biostatistics, School of Public Health, Anhui Medical University, 81 Meishan Road, Hefei 230032, Anhui, China;; 4 The Second Clinical School of Medicine, Anhui Medical University, 81 Meishan Road, Hefei 230032, Anhui, China

**Keywords:** Anthropometric indicator, osteoporosis, mendelian randomization, bone mineral density, bone fractures, MVMR analysis

## Abstract

**Introduction:**

Although observational studies have suggested potential associations between Anthropometric Indicators (AIs), Osteoporosis (OP), and bone fractures, the causal links are still scarce. This study aimed to comprehensively evaluate the causal relationships between five AIs and site- and age-specific Bone Mineral Density (BMD), as well as bone fractures and falls.

**Methods:**

Genetic exposure data for AIs and outcome data for BMD, bone fractures, and falls were retrieved from various genetic consortia. Genome-wide associations of Single-Nucleotide Polymorphisms (SNPs) were selected as Instrumental Variables (IVs) to infer causal effects in univariable and multivariable Mendelian Randomization (MR) analyses.

**Results:**

The results of primary univariable MR analyses revealed that a per-unit increase in Hip Circumference (HC) was causally associated with a lower risk of bone fractures (Inverse Variance Weighting (IVW) method: OR = 0.80, 95% CI: 0.74, 0.88; *P* = 1.73×10^-06^). After adjusting for Body Mass Index (BMI), smoking, and alcohol consumption, further Multivariable Mendelian Randomization (MVMR) analysis supported the presence of causal effects of HC on the decreased risk of fractures. Nevertheless, no causal associations were found between Neck Circumference (NC), Waist Circumference (WC), Waist-to-Hip Ratio (WHR), or Intracranial Volume (ICV) and site/age-specific BMD or the risk of bone fractures or falls.

**Discussion:**

Genetic MR analysis establishes HC as an independent protective factor against fractures, resolving prior observational evidence. Limitations include European ancestry bias.

**Conclusion:**

This study reveals an independent causal association between HC and a lower risk of bone fractures, suggesting that an appropriate increase in HC is beneficial for the prevention of OP.

## 1 INTRODUCTION

Osteoporosis (OP) is a systemic metabolic bone disease characterized by accelerated bone resorption and a decline in Bone Mineral Density (BMD) and strength, resulting in enhanced bone fragility and a higher likelihood of bone fractures [1]. The global prevalence of OP is estimated to be approximately 19.7%, with a notable increase observed as age advances [2]. OP has become one of the most prevalent metabolic disorders, representing a leading cause of morbidity and mortality in geriatric populations. To date, the etiology of OP remains poorly understood. Mounting evidence has demonstrated that aging, parathyroid and thyroid dysfunction, smoking, alcohol consumption, oxidative stress (accumulation of Reactive Oxygen Species (ROS) and Reactive Nitrogen Species (RNS)), and genetic predisposition, and interleukins and other cytokines secreted by leukocytes included tissue macrophages and monocytes in response to various bacterial infections contribute to the pathogenesis of OP [3-6]. An increasing body of research underscores the crucial role of physical attributes in assessing OP and predicting future fracture risk [7].

Anthropometric Indicators (AIs), including Waist Circumference (WC), height, weight, and Hip Circumference (HC), are non-invasive quantitative measurements of the body [8]. In recent years, numerous observational studies have explored relationships between various AIs and BMD and bone fractures [9, 10]. However, some studies have reported conflicting results [11, 12]. A prior study based on the National Health and Nutrition Examination Survey (NHANES) revealed a positive association between WC and BMD [13, 14]. In contrast, another study found that WC was negatively correlated to BMD in different age and Body Mass Index (BMI) groups [15]. These inconsistencies may be attributed to differences in study populations, methodologies, and confounding factors such as age, sex, and hormonal status. Given the reported inconsistencies and the lack of randomized controlled trials, the causal role of AIs in BMD and fractures risk remains uncertain and warrants further investigation.

Mendelian Randomization (MR) is a novel epidemiological tool that utilizes genetic variants as Instrumental Variables (IVs) to infer causal associations among exposures and outcomes [16]. By leveraging the random allocation of genetic variants from parents to offspring, MR can overcome the limitations of traditional observational designs and minimize bias from potential confounders and reverse causality [17, 18]. In the present study, Univariable MR (UVMR) approaches were utilized to infer the causality between five AIs, including Neck Circumference (NC), WC, HC, Waist-Hip Ratio (WHR), Intracranial Volume (ICV), and site/age-specific BMD, as well as the risk for bone fractures and falls. Moreover, Multivariable MR (MVMR) analysis was performed to assess the direct causality after accounting for BMI, smoking status, and alcohol consumption.

## 2 METHODS

### 2.1 Study Design

In MR analysis, genetic variants, typically Single-Nucleotide Polymorphisms (SNPs), are defined as IVs to estimate the causal effects of modifiable exposure on outcomes. Three crucial assumptions must be satisfied for IVs to be valid [19]. (1) Relevance assumption, IVs must demonstrate a strong association with the exposure factor [20]. (2) Exclusion restriction assumption, the identified IVs should affect the outcome only through exposure rather than other factors [21]. (3) Independence assumption, IVs must be independent of confounders for exposure-outcome associations [22].

The current study had a two-phase design. In the first phase, UVMR analysis was conducted to investigate the causal relationships between five AIs and five site-specific BMD groups (Lumbar Spine BMD (LS-BMD), Forearm BMD (FA-BMD), Femoral Neck BMD (FN-BMD), Estimated BMD (eBMD) from quantitative heel ultrasounds, and Total Body BMD (TB-BMD)). In addition, five age-specific BMD groups (age ≤ 15, 15 < age ≤ 30, 30 < age ≤ 45, 45 < age < 60, and age ≥ 60), and the risk for bone fractures and falls were analyzed. Then, MVMR analysis was performed to verify causality after adjusting for some confounders, such as BMI, smoking, and alcohol consumption. (Fig. (**1**)) illustrates the study design.

### 2.2 Data Sources and IVs Selection

Five types of AIs were selected as exposures, such as HC, WC, NC, WHR, and ICV. Summary statistics from Genome-Wide Association Study (GWAS) for HC and WC were obtained from exome-wide association studies comprising 19,292 Finnish participants [23]. Summary GWAS data for NC reported by Liu *et al.*, which included 15,090 individuals [24]. In addition, WHR (BMI-adjusted) data were obtained from a large genetic consortium comprising 288,492 individuals [25]. Genetic summary data for ICV were sourced from another large consortium, which includes 32,438 adults [26]. All the exposure datasets were generated from individuals of European ancestry. BMD was used as an outcome to represent the phenotype of OP. Due to the varied BMD among the different body sites and age subgroups, the genetic summary data regarding five site-specific BMD (LS-BMD, FA-BMD, FN-BMD, eBMD and TB-BMD) and five age-specific BMD (age ≤ 15, 15 < age ≤ 30, 30 < age ≤ 45, 45 < age < 60 and age ≥ 60) were derived from the three large GWAS analysis consortiums based on the reports of Zheng *et al.*, Kemp *et al.* and Medina-Gomez *et al.*, respectively [27-29], where the FN-BMD, LS-BMD, FA-BMD, TB-BMD and age-specific BMD were measured *via* Dual Energy X-Ray Absorptiometry (DEXA), and the eBMD was detected *via* quantitative ultrasonography. Considering the close relationships between OP and fractures and falls, summary statistics for fractures and falls were obtained from two publicly available GWAS by Trajanoska *et al.* [30, 31]. All genetic summary-level data were downloaded from the Genetic Factors for Osteoporosis Consortium (GEFOS).

A stringent threshold of *P* < 5 × 10^−08^ was initially set for selecting AI-associated SNPs as IVs. However, since the majority of SNPs failed to meet this criterion, the criterion was relaxed to *P* < 1 × 10^-05^ for IVs selection. Moreover, to avoid possible bias, SNPs with strong Linkage Disequilibrium (LD) (*R*^2^ < 0.001, window size = 5000 kb) were removed. This study revealed a diverse set of genetically associated loci, including 7 HC-associated SNPs, 6 WC-associated SNPs, 4 NC-associated SNPs, 42 WHR-associated SNPs, and 39 ICV-associated SNPs (Supplementary Table **1**).

### 2.3 Statistical Analysis

#### 2.3.1 UVMR Analysis

The causal effects of HC, WC, NC, WHR, and ICV (per standard deviation (SD)) on BMD and the risk for fractures and falls were estimated using Inverse Variance Weighting (IVW), Weighted Median (WM), Weighted Model, and MR Egger methods. These approaches provided optimal precision when all instruments were valid and reliable. *F*-statistics for each SNP were calculated to assess weak instrument bias, with validity generally assumed for *F*-statistics > 10, where *F = R^2^ × (N − 2) / (1 − R^2^)*, and *R^2^* represents the proportion of exposure variance explained by each instrument [32]. To control for multiple testing, the Bonferroni correction was performed with a statistical significance of *P* < 8.3 × 10^-04^ (0.05 / 60; 5 exposures and 12 outcomes).

#### 2.3.2 MVMR Analysis

Smoking, alcohol consumption, and BMI are major confounding factors associated with both the 5 AIs used as exposure and the outcomes of BMD, bone fractures, and falls. Therefore, MVMR analysis was utilized to determine the independent effects of AIs on BMD and the risk of fractures and falls. In MVMR analysis, the MR Pleiotropy RESidual Sum and Outlier test (MR-PRESSO) model was employed to address horizontal pleiotropy in causal effects, estimate heterogeneity, and exclude potential outliers. Statistical significance was set at *P* < 0.05.

#### 2.3.3 Sensitivity Analysis

To evaluate the stability and reliability of the causal effects, as well as to reduce the presence of multi-effectiveness and identify potential outliers. The leave-one-out method was employed in sensitivity analysis to determine if individual SNPs disproportionately impacted the MR estimates. To better control the multi-effectiveness of the study, the SNPs with *P*-values less than 0.05 / N were removed from the results (*N* is the number of significant SNPs). Statistical efficacy (power) was calculated *via* the online tool “mRnd.” Statistical analyses and data visualization were performed using R programming software (Version 4.0.3).

## 3 RESULTS

A total of 98 SNPs associated with AIs were identified from 4 independent GWASs, including 7 HC-associated SNPs, 6 WC-associated SNPs, 4 NC-associated SNPs, 42 WHR-associated SNPs, and 39 ICV-associated SNPs. The *F*-statistics for all 98 AI-associated SNPs exceeded 10, indicating a low likelihood of weak IVs among the included SNPs. Moreover, the selected independent variables accounted for approximately 8.04% (HC), 8.51% (WC), 6.57% (NC), 5.11% (WHR), and 4.30% (ICV) of the phenotypic variance. The results of sample overlap analysis indicated a low degree of overlap between exposure and outcome, with overlap rates within the datasets being less than 10% (Supplementary Fig. **1**).

### 3.1 Causal Effects of AIs on Site/Age-Specific BMD

The IVW method yielded a positive association between NC and FN-BMD (β = 0.06, 95% CI: 0.01, 0.11, *P* = 0.024). However, accounting for potential false positives due to multiple testing, the causal effects of NC on FN-BMD were not statistically significant after the Bonferroni correction (*P* > 8.3 × 10^-04^). In terms of causal associations between AIs and site-specific BMD, no causal relationships were observed between 5 AIs (HC, WC, NC, WHR, and ICV) and site-specific BMD (FA-BMD, LS-BMD, FN-BMD, eBMD, and TB-BMD) (Fig. **2** and Supplementary Table **2**).

AIs: Anthropometric indicators; GWAS: Genome-wide association analysis; MR: Mendelian randomization; IVs: Instrumental variables; SNPs: Single nucleotide polymorphisms; HC: Hip circumference; WC: Waist circumference; NC: Neck circumference; WHR: Waist‒hip ratio; ICV: Intracranial volume; BMD: Bone mineral density; FA: Forearm; FN: Femoral neck; LS: Lumbar spine; eBMD: Estimated from quantitative heel ultrasounds; TB: Total body BMD; N. SNPs: number of single-nucleotide polymorphisms; CI: Confidence interval; IVW: Inverse Variance Weighting.

To understand how AIs affect BMD across different age groups, the causal relationships between these indices and five subgroups of age-specific TB-BMD were examined. Specifically, TB-BMD was analyzed in individuals aged ≤ 15, 15 < age ≤ 30, 30 < age ≤ 45, 45 < age < 60, and age ≥ 60. After Bonferroni correction, no significant causal associations were observed between HC, WC, NC, WHR, or ICV and the five age-specific TB-BMD groups (Fig. **3** and Supplementary Table **3**).

### 3.2 Causal Associations of AIs on the Risk of Bone Fractures and Falls

The results of the IVW method yielded a significant causal relationship between HC and the risk of fractures. Specifically, a unit increase in HC was inversely correlated with the likelihood of risk of bone fractures (OR_IVW_ = 0.80, 95% CI: 0.74, 0.88, *P* = 1.73 × 10^-06^) (Fig. **4**), which was also supported by the WM method (OR_WM_ = 0.77, 95% CI: 0.68, 0.87, *P* = 2.76 × 10^-05^) (Supplementary Table **4**). Sensitivity analysis using the leave-one-out method indicated that the causal results were robust and not significantly influenced by any single SNP (Fig. **5**). Considering the potential confounders of traditional risk factors of BMI, smoking, and alcohol consumption during the causal inference of HC and bone fractures, an additional MVMR analysis was conducted to determine the direct causal associations of HC on the risk of bone fractures, and the results indicated that after adjusting for BMI, smoking, and alcohol consumption, the relationships between a genetically determined HC increase and a lower risk of bone fractures remained significant (OR = 0.88, 95% CI: 0.79, 0.98, *P* = 0.020) (Fig. **6**), suggesting that higher HC may act as a protective role in reducing the risk of bone fractures.

Although there was a causal effect of NC on the risk of bone fractures (OR_IVW_ = 0.96, 95% CI: 0.93, 0.99, *P* = 0.037), the results of the WM method did not support causality between NC and bone fractures (OR_WM_ = 0.96, 95% CI: 0.992, 1.01, *P* = 0.116). Moreover, no causal associations were observed between WC, WHR, or ICV and the risk of bone fractures. In terms of causal inference between AIs and falls, the results of UVMR analysis did not reveal causal effects of HC, WC, NC, WHR, or ICV on the risk of falls (Fig. **4** and Supplementary Table **4**).

## 4 DISCUSSION

In the present study, two phases of MR analyses were performed to comprehensively evaluate the causal associations between five anthropometric indices (HC, WC, NC, WHR, and ICV) and site- and age-specific BMD, as well as the risk of bone fractures and falls. These findings indicated that genetically determined HC increase was causally linked with a lower risk of bone fractures, even after adjusting for the traditional risk factors for smoking, alcohol consumption, BMI, and physical inactivity. A larger HC typically reflects increased gluteal muscle mass and subcutaneous fat deposition in the hip and thigh regions, which can serve as mechanical protection during falls [33, 34]. The cushioning effect of soft tissues surrounding the hip joint has been proposed as a critical factor in reducing impact force upon falling, thereby lowering the likelihood of hip fractures. Additionally, HC may be indicative of a broader skeletal structure, including greater femoral neck width and cortical bone thickness. These anatomical characteristics are associated with enhanced bone strength and resistance to fractures, further supporting the protective role of HC in skeletal fragility. HC is closely related to gluteal muscle mass, which plays a crucial role in balance, postural stability, and gait efficiency [35]. Increased muscle mass in the hip region has been associated with a lower likelihood of falls, a primary risk factor for fractures in older adults. Muscle weakness, particularly in the lower extremities, is a well-recognized predictor of fall-related fractures. Thus, a larger HC, reflecting higher muscle mass, may contribute to better neuromuscular control and stability, reducing both fall risk and subsequent fracture incidence.

Several observational studies have investigated the association between HC and the risk of bone fractures. A prior prospective study in a cohort of 30,652 US postmenopausal women reported a strong inverse association between HC and hip fractures, where the quintiles of HC were associated with a reduced risk of hip fractures after adjusting for age and BMI [9]. Similarly, in middle-aged women, the findings of Klingberg implied that, compared with the lowest quintile of HC, the higher quintile of HC was negatively correlated with the risk of hip fractureswhen controlling for age [10]. Nevertheless, contradictory results have also been reported in some studies, where Meyer *et al*. reported no significant association between HC and hip fractures in either male or female subjects after controlling for BMI [36]. A previous MR study, based on summary data from a European GWAS, reported results inconsistent with these findings, which showed that HC-adjBMI was not associated with the risk of bone fractures [12]. This discrepancy can be explained as follows. Firstly, the data for HC-adjusted BMI were corrected for BMI before the GWAS. However, the selection of HC-associated SNPs may not fully capture the variations in HC that are independent of BMI. In addition, the criteria for the selection of independent SNPs were not strict, and the exclusion of SNPs with strong LD was set as a window size of 1000 kb and *R^2^* < 0.1, which might result in potential weak IVs bias. Moreover, only five HC-associated SNPs were included for causal inference, with a limited variance in phenotype explained, which may have reduced statistical power in detecting a true causal effect between HC and bone fractures risk. Furthermore, variations in study populations and sample sizes may also contribute to inconsistent findings.

Several limitations of this study should be acknowledged. Firstly, MR relies on three core assumptions for valid causal inference. (1) the relevance assumption, which requires that genetic instruments are strongly associated with the exposure. (2) The exclusion restriction assumption, which states that genetic instruments influence the outcome solely through the exposure and not *via* alternative pathways. Furthermore, (3) the independence assumption, which requires that genetic instruments are independent of confounders affecting the exposure-outcome relationship. While the first assumption can be evaluated using the *F*-statistic (where values >10 indicate sufficient strength), the latter two are inherently unverifiable in observational settings, potentially introducing bias into these findings. Secondly, the absence of genetic summary statistics for other AIs, such as head or calf circumference, limits the ability to fully elucidate the impact of these measures on BMD and the risk of fractures or falls. Thirdly, the lack of individual demographic data for AIs prevents us from determining causal relationships between sex-/age-specific AIs and BMD or fractures. Fourtly, the use of a *P* < 1 × 10^-05^ for selecting IVs may have included SNPs with weaker associations to the exposure, potentially increasing the risk of false positive results. Finally, since the GWAS summary data used in this study were derived exclusively from European populations, the generalizability of these findings to other ethnic groups remains uncertain.

Nevertheless, this study has several strengths. Although one previous MR study focused on the relationships between height, BMI, WHR-adjusted BMI, WC-adjusted BMI, and HC-adjusted BMI and BMD/bone fractures, evidence regarding the causal effects of other AIs, such as NC and ICV, on BMD, bone fractures, and falls remains limited. To the best of our knowledge, this is the first study that uses both UVMR and MVMR to comprehensively evaluate the causal associations of HC, WC, NC, WHR, and ICV with BMD and the risk of bone fractures and falls. Moreover, given the complex relationships between age-related changes in TB-BMD over the life course, we investigated the causal effects of these AIs on age-specific TB-BMD. This approach provides deeper insights into the role of genetic determinants of anthropometric traits in BMD changes across different age groups.

## CONCLUSION

Overall, this study provides robust evidence supporting the causal effect of genetically determined HC on reducing fracture risk, independent of confounding factors such as BMI, smoking, and alcohol consumption. These findings suggest that HC may play a protective role in improving bone health and preventing fractures, offering new insights into its potential as a biomarker and therapeutic target for bone-related diseases. Further research is warranted to investigate underlying mechanisms and assess whether interventions aimed at modulating HC levels could facilitate the development of personalized strategies for fracture prevention. Additionally, translating these insights into actionable applications holds promise for improving clinical outcomes related to bone health.

## Figures and Tables

**Fig. (1):**
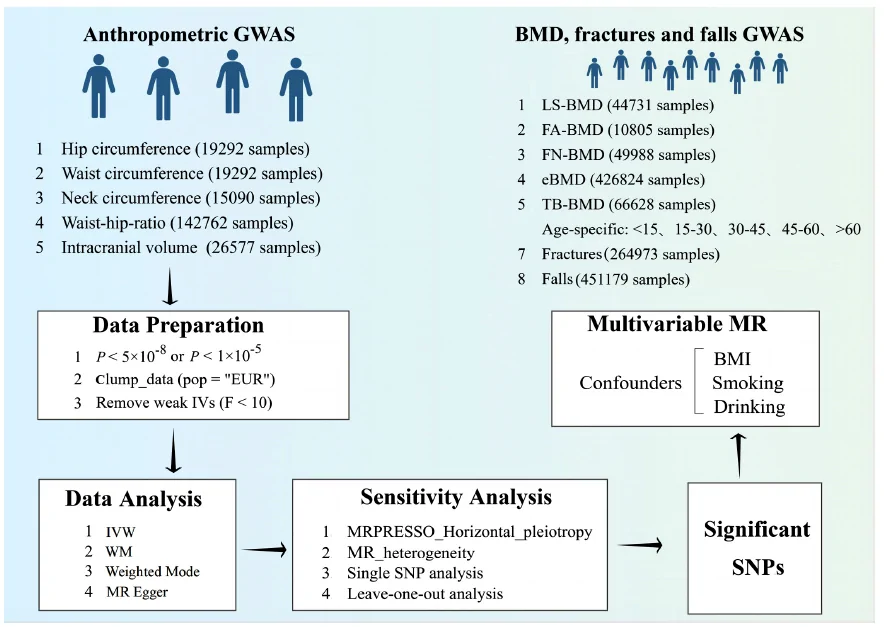
Flowchart of the MR study design evaluating AIs and BMD, as well as fractures and falls. **Abbreviations:** AIs: Anthropometric indicators; GWAS: Genome-wide association analysis; MR: Mendelian randomization; IVs: Instrumental variables; SNPs: Single nucleotide polymorphisms; BMI: Body mass index; BMD: Bone mineral density; FA: Forearm; FN: Femoral neck; LS: Lumbar spine; eBMD: Estimated from quantitative heel ultrasounds; TB: Total body BMD; IVW: Inverse Variance Weighting; WM: Weighted Median.

**Fig. (2):**
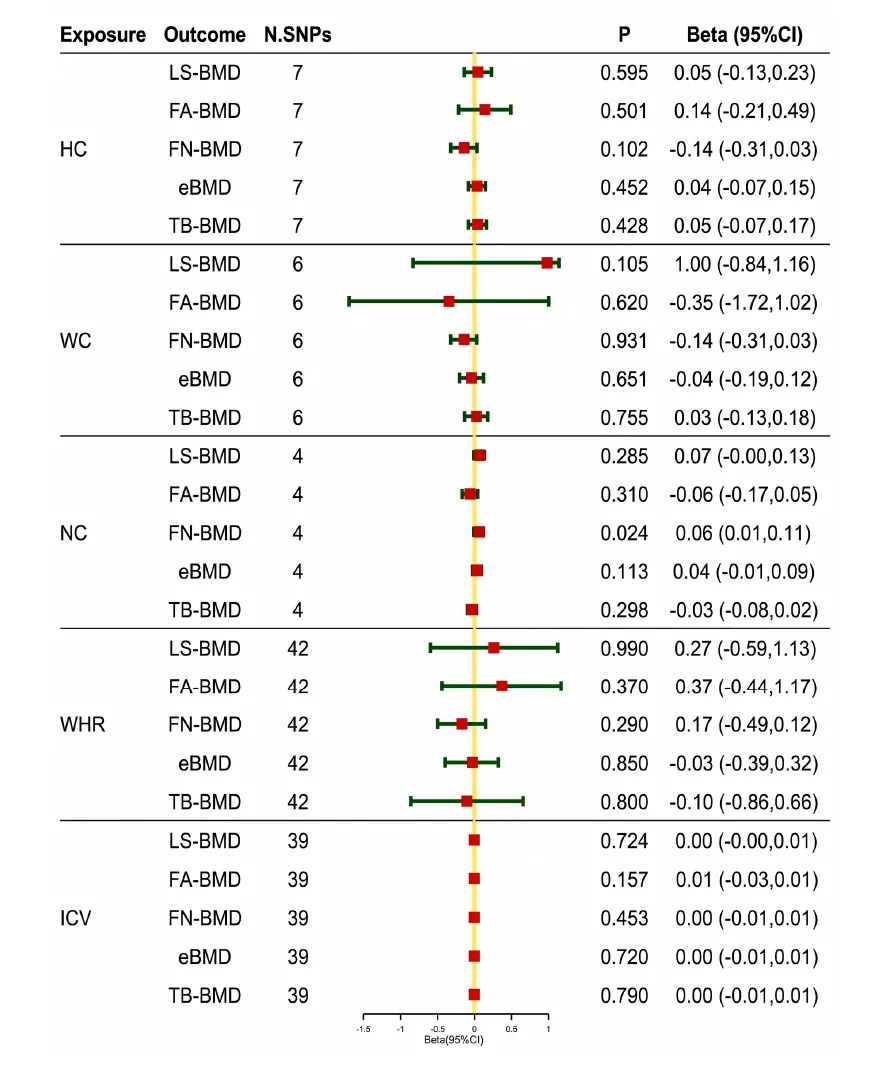
Causal relationships between AIs and site-specific BMD *via* the IVW analysis. **Abbreviations:** AIs: Anthropometric indicators; GWAS: Genome-wide association analysis; MR: Mendelian randomization; IVs: Instrumental variables; SNPs: Single nucleotide polymorphisms; HC: Hip circumference; WC: Waist circumference; NC: Neck circumference; WHR: Waist‒hip ratio; ICV: Intracranial volume; BMD: Bone mineral density; FA: Forearm; FN: Femoral neck; LS: Lumbar spine; eBMD: Estimated from quantitative heel ultrasounds; TB: Total body BMD; N. SNPs: number of single-nucleotide polymorphisms; CI: Confidence interval; IVW: Inverse Variance Weighting.

**Fig. (3):**
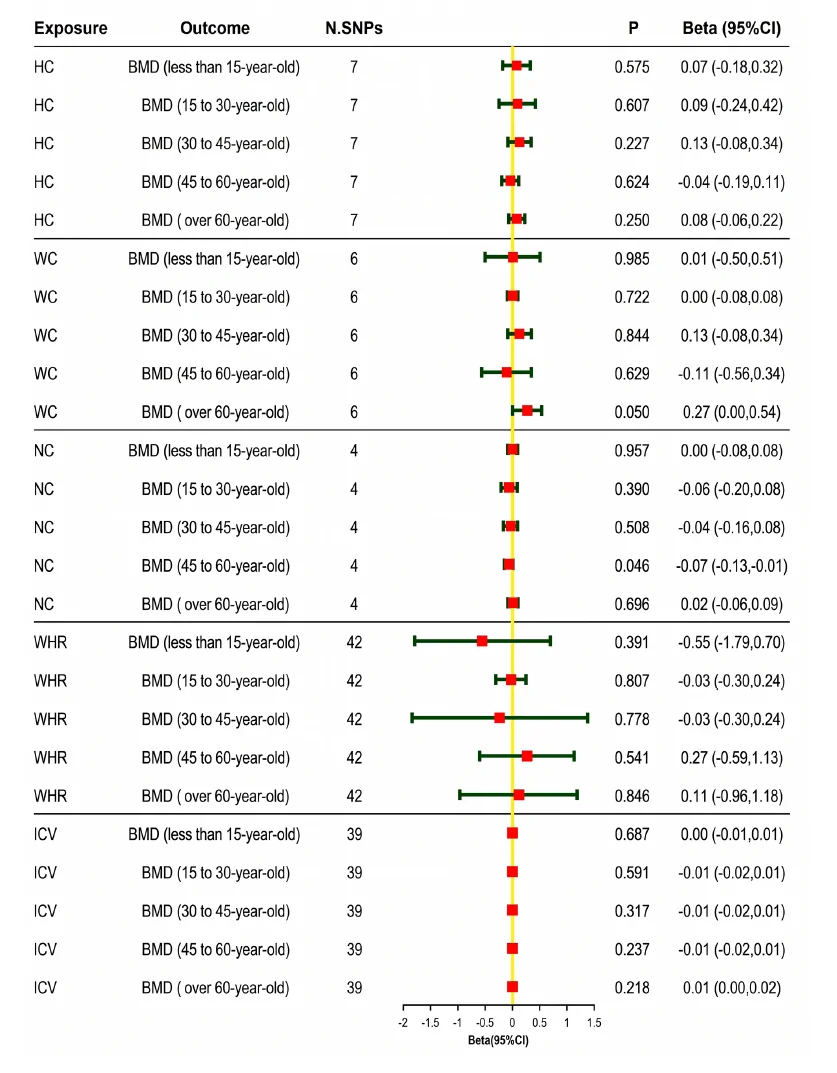
Causal relationships between AIs and age-specific BMD *via* the IVW analysis. **Abbreviations:** AIs: Anthropometric indicators; HC: Hip circumference; WC: Waist circumference; NC: Neck circumference; WHR: Waist‒hip ratio; ICV: Intracranial volume; BMD: Bone mineral density; CI: Confidence interval; N. SNPs: Number of single-nucleotide polymorphisms; IVW: Inverse Variance Weighting.

**Fig. (4):**
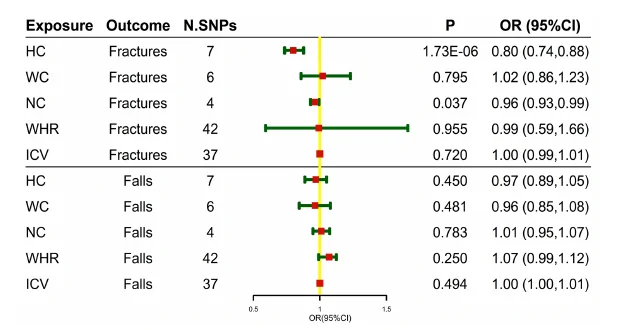
Causal relationships of AIs on the risk of bone fractures and falls *via* the IVW analysis. **Abbreviations:** AIs: Anthropometric indicators; HC: Hip circumference; WC: Waist circumference; NC: Neck circumference; WHR: Waist‒hip ratio; ICV: Intracranial volume; BMD: Bone mineral density; CI: Confidence interval; N. SNPs: Number of single-nucleotide polymorphisms; OR: Odds ratio; IVW: Inverse Variance Weighting.

**Fig. (5):**
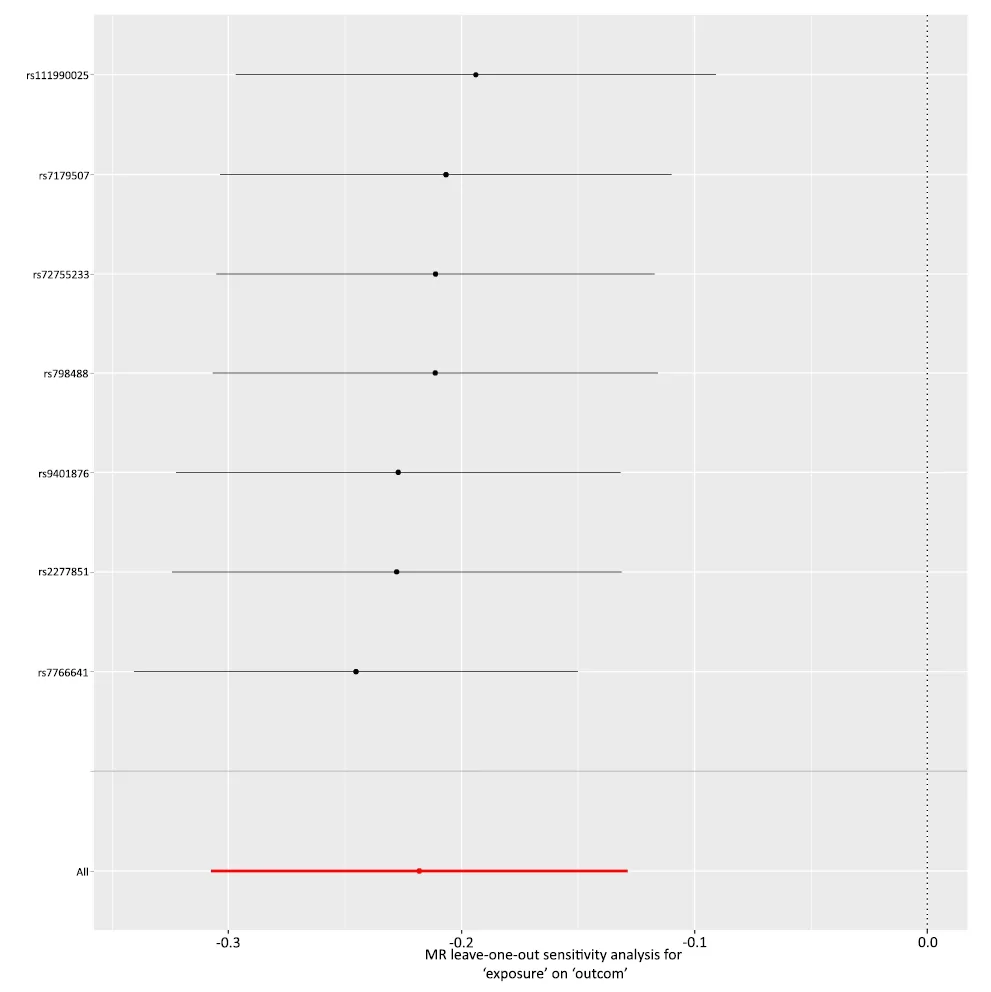
Causal relationship of HC on the risk of bone fractures by using a leave-one-out analysis. **Abbreviation:** HC: Hip Circumference.

**Fig. (6):**
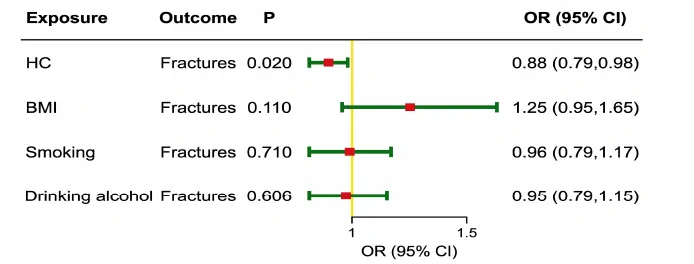
Causal relationships between HC and the risk of bone fractures after accounting for BMI, smoking, and alcohol consumption. **Abbreviations:** BMI: Body Mass Index; HC: Hip Circumference; OR: Odds Ratio.

## Data Availability

The data and materials that support the findings of this study are available from public datasets, which can be found in the NHGRIEBI GWAS Catalog.

## LIST OF ABBREVIATIONS

AIs: Anthropometric Indicators
BMI: Body Mass Index
BMD: Bone Mineral Density
CI: Confidence Interval
eBMD: Estimated Bone Mineral Density
FA-BMD: Forearm Bone Mineral Density
FN-BMD: Femoral Neck Bone Mineral Density
GWAS: Genome-Wide Association Study
HC: Hip Circumference
ICV: Intracranial Volume
IVs: Instrumental Variables
IVW: Inverse Variance Weighting
LD: Linkage Disequilibrium
LS-BMD: Lumbar Spine Bone Mineral Density
MR: Mendelian Randomization
MR-PRESSO: Mendelian randomization, Pleiotropy, RESidual Sum and Outlier Test
MVMR: Multivariable Mendelian Randomization
NC: Neck Circumference
NHANES: The National Health and Nutrition Examination Survey
N. SNPs: Number of Single-Nucleotide Polymorphisms
OP: Osteoporosis
OR: Odds Ratio
RNS: Reactive Nitrogen Species
ROS: Reactive Oxygen Species
TB-BMD: Total Body Bone Mineral Density
UVMR: Univariable Mendelian Randomization
WC: Waist Circumference
WHR: Waist-To-Hip Ratio
WM: Weighted Median
SNPs: Single-Nucleotide Polymorphisms
