# Supplementary material for: Associations of Anthropometric Indicators with Bone Mineral Density and the Risk of Bone Fractures and Falls: A Large-Scale Genetic Correlation Study
Source: Endocr Metab Immune Disord Drug Targets. 2025 Jul 18;26:E18715303369995. doi: 10.2174/0118715303369995250702111723 (PMC13670186; doi:10.2174/0118715303369995250702111723)
Supplement: Supplementary file 1 [file EMIDDT-26-E18715303369995_SD1.pdf]

Supplementary Material

Associations of Anthropometric Indicators with Bone Mineral Density and the Risk of Bone Fractures and Falls: A Large-Scale Genetic Correlation Study

Xiao Hu[1,2,#] , Zhao-Xing Gao[2,3,#] , Yan-Yu Zhu[2,3,#] , Sheng Li[1,2] , Wen-Wen Ding[4], Hai-Feng Pan[2,3,*] and Peng Wang[1,2,*]

[1]Department of Health Promotion and Behavioral Sciences, School of Public Health, Anhui Medical University, 81 Meishan Road, Hefei 230032, Anhui, China; [2]Institute of Kidney Disease, Inflammation & Immunity Mediated Diseases, the Second Hospital of Anhui Medical University, Hefei 230032, Anhui, China; [3]Department of Epidemiology and Biostatistics, School of Public Health, Anhui Medical University, 81 Meishan Road, Hefei 230032, Anhui, China; [4]The Second Clinical School of Medicine, Anhui Medical University, 81 Meishan Road, Hefei 230032, Anhui, China

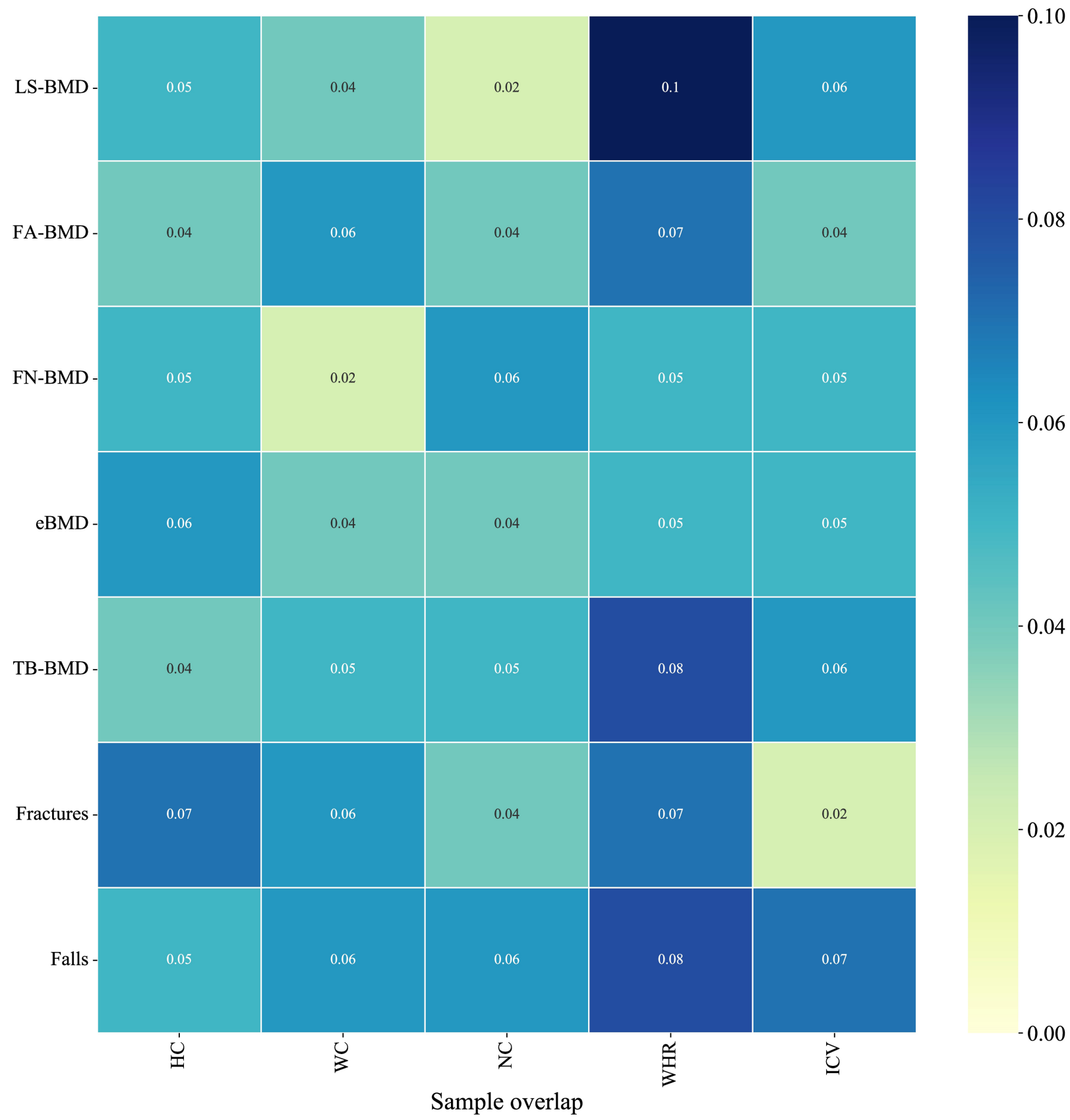

Supplementary Figure 1 Sample overlap between AIs and BMD, as well as fractures and falls.
AIs: Anthropometric indicators; HC: Hip circumference; WC: Waist circumference; NC: Neck circumference; WHR: Waist–hip ratio; ICV: Intracranial volume; BMD: Bone mineral density; FA: Forearm; FN: Femoral neck; LS: Lumbar spine; eBMD: Estimated from quantitative heel ultrasounds BMD; TB: Total body BMD

Table S1. Single nucleotide polymorphisms (SNPS) associated with anthropometric indicators (used as IVs of Mendelian randomization).

SNP	Chr	Position	Effect allele	Other allele	EAF	Effect	Standard error	*P* Value	n	Phenotype	F-statistic	Variance explained (%)
rs111990025	3	33543195	C	T	0.01307	0.219	0.048	5.48E-06	19292	HC	20.664	0.107
rs2277851	16	4414987	G	A	0.4843	0.054	0.011	1.96E-06	19292	HC	22.652	0.117
rs7179507	15	84473531	G	A	0.32153	0.059	0.012	7.30E-07	19292	HC	24.549	0.127
rs72755233	15	100692953	G	A	0.12471	-0.070	0.016	7.57E-06	19292	HC	20.058	0.104
rs7766641	6	26184102	G	A	0.44145	-0.057	0.013	5.82E-06	19292	HC	20.546	0.106
rs798488	7	2802522	T	C	0.37828	-0.055	0.011	7.11E-07	19292	HC	24.605	0.127
rs9401876	6	126669479	C	A	0.43801	0.050	0.011	2.54E-06	19292	HC	22.158	0.115
rs2302580	4	8608634	C	T	0.40559	-0.060	0.011	6.35E-08	19292	WC	29.253	0.151
rs173964	5	55809465	A	G	0.32624	-0.056	0.011	9.49E-07	19292	WC	24.055	0.125
rs1966265	5	176516631	G	A	0.24585	0.072	0.012	6.44E-09	19292	WC	33.728	0.175
rs7040769	9	78506187	T	C	0.19295	0.059	0.013	8.15E-06	19292	WC	19.916	0.103
rs72755233	15	100692953	G	A	0.12474	-0.096	0.016	8.81E-10	19292	WC	37.591	0.194
rs4551339 1	19	54631381	G	A	0.28602	0.0537	0.012	7.90E-06	19292	WC	19.957	0.103
rs122308886	12	52268213	T	C	0.8943	-0.282	0.058	1.18E-06	15090	NC	23.637	0.156
rs4557101	3	73540618	T	C	0.6333	-0.1529	0.0343	8.08E-06	15090	NC	19.869	0.132
rs150835180	2	202236630	A	G	0.9493	-0.3659	0.0821	8.27E-06	15090	NC	19.860	0.131
rs9923147	16	53801549	T	C	0.5918	-0.2026	0.0338	2.00E-09	15090	NC	35.924	0.238
rs1011731	1	172300000	G	A	0.482	-0.035	0.004	2.65E-20	334966	WHR	85.237	0.025

rs1034405	3	50597092	G	A	0.167	-0.020	0.004	1.05E-06	323449	WHR	23.834	0.007
rs11057401	12	124400000	T	A	0.250	-0.052	0.006	5.36E-18	335701	WHR	74.744	0.022
rs11755724	6	7118990	A	G	0.291	0.022	0.004	7.90E-09	344369	WHR	33.300	0.010
rs1178979	7	72856430	T	C	0.172	-0.050	0.009	1.47E-07	344369	WHR	27.633	0.008
rs12700667	7	25901639	G	A	0.463	-0.026	0.004	1.02E-11	312513	WHR	46.287	0.015
rs12735613	1	118900000	G	A	0.241	-0.040	0.007	6.42E-08	344369	WHR	29.234	0.008
rs1294421	6	6743149	T	G	0.473	0.037	0.004	2.16E-24	344369	WHR	103.866	0.030
rs13133548	4	89740128	G	A	0.456	0.021	0.004	1.56E-07	344369	WHR	27.516	0.008
rs13326165	3	52532118	A	G	0.197	0.022	0.004	2.26E-09	344369	WHR	35.736	0.010
rs13389219	2	165500000	C	T	0.397	-0.046	0.005	2.60E-18	283807	WHR	76.169	0.027
rs138786	22	35728466	T	C	0.436	-0.019	0.004	1.02E-06	314130	WHR	23.887	0.008
rs143384	20	34025756	A	G	0.439	-0.029	0.004	6.77E-11	344369	WHR	42.583	0.012
rs1443512	12	54342684	A	C	0.339	-0.021	0.004	5.15E-09	344369	WHR	34.131	0.010
rs1966265	5	176500000	G	A	0.229	0.038	0.008	7.07E-07	323449	WHR	24.597	0.008
rs2145270	20	6621685	C	T	0.375	-0.023	0.004	1.15E-10	344369	WHR	41.554	0.012
rs2276853	3	47282303	G	A	0.440	0.017	0.004	3.98E-06	344369	WHR	21.272	0.006
rs2307019	19	49244220	G	A	0.486	-0.021	0.004	1.85E-06	344369	WHR	22.747	0.007
rs2745353	6	127500000	C	T	0.448	0.032	0.004	1.48E-15	322634	WHR	63.663	0.020
rs2925979	16	81534790	T	C	0.290	-0.023	0.003	5.29E-11	344369	WHR	43.066	0.013
rs35349911	6	43785255	C	T	0.479	0.018	0.004	5.97E-06	293210	WHR	20.498	0.007
rs3733526	4	120500000	G	A	0.190	-0.020	0.004	1.13E-07	329480	WHR	28.138	0.009

rs3810818	16	4432029	A	C	0.314	-0.023	0.004	2.63E-09	292436	WHR	35.440	0.012
rs4369779	18	20735408	T	C	0.205	0.017	0.004	4.67E-06	344369	WHR	20.969	0.006
rs459193	5	55806751	A	G	0.394	-0.026	0.004	7.10E-13	319090	WHR	51.517	0.016
rs4823006	22	29451671	A	G	0.454	-0.021	0.004	8.37E-07	344369	WHR	24.270	0.007
rs4846567	1	219800000	G	T	0.223	-0.052	0.007	1.13E-15	344369	WHR	64.198	0.019
rs6465825	7	77416439	T	C	0.378	-0.023	0.005	2.13E-06	344369	WHR	22.473	0.007
rs6487543	12	26438189	G	A	0.460	0.024	0.004	1.16E-09	288851	WHR	37.044	0.013
rs6795735	3	64705365	C	T	0.287	-0.030	0.004	3.84E-12	344369	WHR	48.203	0.014
rs6905288	6	43758873	G	A	0.355	0.037	0.004	7.08E-24	344369	WHR	101.518	0.029
rs718314	12	26453283	A	G	0.354	0.044	0.008	7.34E-08	239062	WHR	28.973	0.012
rs757608	17	59497277	A	G	0.300	-0.024	0.003	2.69E-12	344369	WHR	48.901	0.014
rs7630214	3	52554304	G	C	0.017	-0.027	0.006	7.05E-06	317612	WHR	20.179	0.006
rs797486	13	51221618	C	A	0.144	0.024	0.004	1.70E-08	314130	WHR	31.809	0.010
rs874628	19	18304700	A	G	0.175	0.032	0.007	2.09E-06	344369	WHR	22.513	0.007
rs885389	12	131600000	A	G	0.426	0.019	0.004	6.86E-08	325852	WHR	29.103	0.009
rs893817	15	74229065	G	A	0.354	-0.016	0.004	9.69E-06	323449	WHR	19.572	0.006
rs897453	17	17425631	C	T	0.199	-0.022	0.005	1.76E-06	344369	WHR	22.840	0.007
rs900400	3	156800000	T	C	0.346	-0.031	0.006	2.43E-07	226264	WHR	26.657	0.012
rs984222	1	119500000	C	G	0.500	0.033	0.004	5.65E-17	294612	WHR	70.095	0.024
rs10817699	9	117772582	A	T	0.255	-0.007	0.002	4.23E-06	26577	ICV	21.158	0.080
rs111327216	3	49525218	T	C	0.093	0.050	0.010	1.30E-06	26425	ICV	23.424	0.089
rs11136864	8	531083	A	C	0.781	0.004	0.001	7.52E-06	26577	ICV	20.051	0.075

rs113528138	10	105125451	A	C	0.331	0.012	0.002	2.22E-10	26577	ICV	40.256	0.151
rs11872479	18	46227748	T	C	0.097	0.007	0.002	6.31E-06	26302	ICV	20.393	0.077
rs12452301	17	27862659	T	C	0.180	0.001	0.000	5.54E-06	26577	ICV	20.637	0.078
rs12452834	17	47111739	T	C	0.333	-0.014	0.003	2.70E-06	26577	ICV	22.013	0.083
rs13001657	2	88946611	A	G	0.295	0.018	0.004	6.80E-06	26577	ICV	20.248	0.076
rs13015080	2	217384482	A	G	0.294	0.007	0.002	5.75E-06	26577	ICV	20.565	0.077
rs1351394	12	66351826	T	C	0.492	0.003	0.001	1.23E-08	26577	ICV	30.215	0.114
rs145768812	8	108938468	C	G	0.897	0.001	0.000	3.33E-06	25297	ICV	21.611	0.085
rs151205935	16	87685612	A	G	0.048	-0.029	0.006	3.14E-06	26347	ICV	21.723	0.082
rs16974131	13	110058348	T	C	0.772	-0.011	0.003	7.66E-06	26577	ICV	20.024	0.075
rs17284573	5	82929576	T	C	0.932	-0.015	0.003	1.16E-07	26577	ICV	28.077	0.106
rs17650496	6	127270764	A	G	0.941	-0.041	0.008	4.33E-07	22969	ICV	25.541	0.111
rs17689824	17	43904397	T	C	0.211	-0.022	0.003	6.61E-14	16712	ICV	56.168	0.335
rs181259205	1	51238213	T	C	0.027	-0.003	0.001	7.09E-06	26287	ICV	20.168	0.077
rs1936793	6	127424842	T	C	0.698	-0.028	0.005	2.57E-10	26577	ICV	39.977	0.150
rs199513	17	44856932	A	G	0.201	-0.013	0.001	4.73E-21	26577	ICV	88.636	0.332
rs2253310	6	108888593	C	G	0.377	-0.016	0.003	3.12E-10	26577	ICV	39.599	0.149
rs2330919	7	43673567	T	G	0.732	0.001	0.000	8.59E-08	26577	ICV	28.663	0.108
rs2583930	12	66246181	A	G	0.855	0.005	0.001	1.52E-06	26577	ICV	23.115	0.087

rs28360588	6	109199213	T	C	0.651	-0.020	0.005	5.35E-06	26577	ICV	20.710	0.078
rs35989701	3	190671225	A	C	0.625	0.002	0.000	4.97E-07	25730	ICV	25.269	0.098
rs3817176	3	141712780	T	C	0.153	0.014	0.003	1.85E-06	26577	ICV	22.742	0.086
rs4773039	13	109951018	A	G	0.842	-0.016	0.004	5.74E-06	26262	ICV	20.574	0.078
rs62275162	3	156572571	A	G	0.047	-0.015	0.003	1.65E-06	26577	ICV	22.962	0.086
rs6736289	2	10195540	C	G	0.784	-0.050	0.010	6.83E-07	26455	ICV	24.659	0.093
rs72888453	2	183876732	A	G	0.865	-0.014	0.003	1.77E-06	26577	ICV	22.828	0.086
rs7811609	7	32930597	T	C	0.386	-0.005	0.001	3.55E-06	26577	ICV	21.491	0.081
rs79367267	1	243689543	A	C	0.147	0.021	0.004	2.17E-06	26455	ICV	22.437	0.085
rs79617314	7	50820013	A	C	0.796	-0.030	0.007	6.84E-06	26577	ICV	20.239	0.076
rs8027539	15	31632806	A	G	0.646	-0.003	0.001	1.28E-06	26577	ICV	23.463	0.088
rs80343768	12	79609654	A	C	0.886	0.034	0.008	6.66E-06	22935	ICV	20.284	0.088
rs843557	4	121678048	C	G	0.733	-0.028	0.006	2.99E-06	26577	ICV	21.817	0.082
rs853974	6	127068983	T	C	0.263	0.003	0.000	2.11E-17	26577	ICV	72.041	0.270
rs9419939	10	104547610	A	G	0.194	0.005	0.001	2.43E-08	26577	ICV	28.556	0.107
rs9555539	13	109670272	T	C	0.148	-0.014	0.003	3.23E-06	26262	ICV	21.667	0.082
rs9811910	3	190670902	C	G	0.081	0.007	0.001	1.99E-09	25947	ICV	35.985	0.139

HC: hip circumference; WC: waist circumference; NC: neck circumference; WHR: waist-to-hip ratio; ICV: intracranial volume; SNP: single nucleotide polymorphism.

Table S2. Two-sample MR analyses of anthropometric traits and different sites-BMD.

Expo-sure	Out-come	SNP (n)	Outcome sample size	IVW			Weighted Methods				MR egger regression				
							Weighted Median		Weighted Mode		MR egger		Intercept		
				Beta (se)	*P* value	*P* heterogenei-ty	Beta (se)	*P* value	Beta (se)	*P* value	Beta (se)	*P* value	Inter-cept	se	*P* value
HC	LS-BMD	7	44731 (GEFO)	0.050 (0.093)	0.595	0.096	0.056 (0.058)	0.336	0.176 (0.141)	0.258	0.393 (0.217)	0.129	0.000	0.018	0.999
HC	FA-BMD	7	10805 (GEFO)	0.142 (0.177)	0.501	0.147	0.159 (0.191)	0.404	0.180 (0.232)	0.482	4.117 (2.682)	0.421	-0.219	0.148	0.234
HC	FN-BMD	7	49988 (GEFO)	-0.142 (0.087)	0.102	0.021	-0.214 (0.078)	0.006	-0.220 (0.111)	0.104	-1.181 (0.264)	0.528	0.003	0.018	0.880
HC	eBMD	7	456824 (GEFO)	0.041 (0.056)	0.452	0.585	0.088 (0.030)	0.003	0.095 (0.042)	0.062	0.149 (0.155)	0.380	-0.007	0.010	0.489
HC	TB-BMD	7	66628 (GEFO)	0.049 (0.062)	0.428	0.035	0.024 (0.059)	0.680	0.058 (0.091)	0.547	0.193 (0.169)	0.304	-0.01	0.011	0.399
WC	LS-BMD	6	44731 (GEFO)	1.000 (0.080)	0.105	1.000	1.000 (0.130)	0.009	1.000 (0.172)	0.007	1.000 (0.405)	0.069	0.101	0.026	1.000
WC	FA-BMD	6	10805 (GEFO)	-0.346 (0.699)	0.620	0.036	-0.416 (0.369)	0.799	-0.405 (0.344)	0.780	-0.746 (0.708)	0.850	0.003	0.016	0.930
WC	FN-BMD	6	49988 (GEFO)	-0.142 (0.087)	0.931	0.021	-0.214 (0.078)	0.006	-0.220 (0.111)	0.104	-0.182 (0.264)	0.528	0.003	0.018	0.880
WC	eBMD	6	456824 (GEFO)	-0.035 (0.077)	0.651	0.010	0.031 (0.034)	0.362	0.035 (0.032)	0.380	-0.266 (1.190)	0.860	0.013	0.068	0.878
WC	TB-BMD	6	66628 (GEFO)	0.025 (0.080)	0.755	0.627	0.028 (0.096)	0.773	0.041 (0.097)	0.715	0.822 (0.963)	0.550	-0.045	0.055	0.559
NC	LS-BMD	4	44731 (GEFO)	0.066 (0.032)	0.285	0.016	-0.076 (0.040)	0.056	-0.081 (0.043)	0.204	-0.037 (0.090)	0.751	-0.007	0.020	0.791
NC	FA-BMD	4	10805 (GEFO)	-0.057 (0.056)	0.310	0.469	-0.078 (0.069)	0.259	-0.097 (0.088)	0.387	-0.239 (0.159)	0.373	0.044	0.036	0.436
NC	FN-BMD	4	49988 (GEFO)	0.061 (0.027)	0.024	0.496	0.042 (0.036)	0.240	0.039 (0.043)	0.460	0.123 (0.077)	0.355	-0.015	0.017	0.549
NC	eBMD	4	456824 (GEFO)	0.041 (0.026)	0.113	4.15E-15	0.001 (0.010)	0.939	-0.003 (0.009)	0.773	0.028 (0.108)	0.817	0.003	0.024	0.914

NC	TB-BMD	4	66628 (GEFO)	-0.026 (0.025)	0.298	0.107	-0.039 (0.022)	0.079	-0.046 (0.025)	0.160	-0.113 (0.085)	0.314	0.020	0.018	0.395
WHR	LS-BMD	42	44731 (GEFO)	0.267 (0.438)	0.990	0.819	0.040 (0.037)	0.985	0.156 (0.646)	0.815	0.161 (0.503)	0.758	0.003	0.008	0.682
WHR	FA-BMD	42	10805 (GEFO)	0.365 (0.410)	0.370	0.117	0.364 (0.998)	0.971	0.333 (0.817)	0.698	0.463 (0.666)	0.518	0.041	0.030	0.567
WHR	FN-BMD	42	49988 (GEFO)	-0.171 (0.163)	0.290	0.480	-0.090 (0.041)	0.976	-0.073 (0.263)	0.789	-0.104 (0.219)	0.651	-0.003	0.007	0.655
WHR	eBMD	42	456824 (GEFO)	-0.034 (0.181)	0.850	7.4E-13	-0.096 (0.843)	0.909	-0.108 (0.111)	0.359	-0.206 (0.192)	0.319	0.005	0.003	0.135
WHR	TB-BMD	42	66628 (GEFO)	-0.098 (0.386)	0.800	0.013	-0.325 (0.725)	0.654	-0.286 (0.417)	0.512	-0.434 (0.401)	0.315	0.010	0.006	0.136
ICV	LS-BMD	39	44731 (GEFO)	0.002 (0.003)	0.724	1.4E-6	0.005 (0.003)	0.054	0.003 (0.003)	0.390	0.005 (0.006)	0.428	-0.002	0.003	0.593
ICV	FA-BMD	39	10805 (GEFO)	-0.011 (0.008)	0.157	0.007	-0.012 (0.009)	0.167	-0.015 (0.010)	0.134	-0.008 (0.016)	0.629	-0.002	0.009	0.827
ICV	FN-BMD	39	49988 (GEFO)	-0.003 (0.004)	0.453	0.002	-0.002 (0.004)	0.684	-0.001 (0.005)	0.912	-0.007 (0.008)	0.373	-0.003	0.004	0.453
ICV	eBMD	39	456824 (GEFO)	-0.002 (0.005)	0.720	1.68E-16	-0.004 (0.004)	0.271	-0.008 (0.004)	0.046	-0.003 (0.011)	0.779	0.003	0.006	0.605
ICV	TB-BMD	39	66628 (GEFO)	-0.001 (0.004)	0.790	4.06E-12	-0.002 (0.003)	0.529	-0.006 (0.004)	0.135	-0.003 (0.008)	0.763	0.001	0.005	0.840

BMD: bone mineral density; FA: forearm; FN: femoral neck; LS: lumbar spine; eBMD: estimated heel BMD; TB:total body BMD; GEFO: Genetic Factors for Osteoporosis Consortium; MR: Mendelian randomization; SNP: single-nucleotide polymorphisms; IVW: inverse-variance weighted.

Table S3. Two-sample MR analyses of anthropometric traits and age-specific BMD.

**Expo-sure**	**Out-come**	**SNP (n)**	**Outcome sample size**	**IVW**			**Weighted Methods**				**MR egger regression**				
							**Weighted Median**		**Weighted Mode**		**MR egger**		**Intercept**		
				**Beta (se)**	***P* value**	***P* hetero-geneity**	**Beta (se)**	***P* val-ue**	**Beta (se)**	***P* val-ue**	**Beta (se)**	***P* val-ue**	**Inter-cept**	**se**	***P* val-ue**
HC	BMD (age ≤ 15)	7	11807 (GEFO)	0.070 (0.125)	0.575	0.121	0.122 (0.134)	0.361	0.111 (0.166)	0.528	0.290 (0.354)	0.450	-0.015	0.023	0.533
HC	BMD (15 < age ≤	7	4180 (GEFO)	0.087 (0.169)	0.607	0.982	0.058 (0.205)	0.776	0.060 (0.279)	0.838	-0.024 (0.442)	0.959	0.008	0.028	0.797

	30)														
HC	BMD (30 < age ≤ 45)	7	9471 (GEFO)	0.130 (0.107)	0.227	0.543	0.157 (0.146)	0.282	0.134 (0.204)	0.534	0.176 (0.295)	0.578	-0.003	0.019	0.875
HC	BMD (45 < age < 60)	7	18735 (GEFO)	-0.037 (0.076)	0.624	0.447	0.044 (0.101)	0.665	0.061 (0.138)	0.673	0.207 (0.201)	0.349	-0.017	0.013	0.244
HC	BMD (age ≥ 60)	7	22435 (GEFO)	0.082 (0.071)	0.250	0.576	0.152 (0.092)	0.099	0.182 (0.120)	0.182	0.245 (0.190)	0.254	-0.011	0.012	0.397
WC	BMD (age ≤ 15)	6	11807 (GEFO)	0.005 (0.260)	0.985	0.119	0.001 (0.218)	0.996	-0.042 (0.254)	0.885	-1.138 (4.354)	0.837	0.065	0.247	0.836
WC	BMD (15 < age ≤ 30)	6	4180 (GEFO)	-0.002 (0.040)	0.722	0.459	0.010 (0.048)	0.834	0.018 (0.058)	0.776	-0.067 (0.152)	0.701	0.015	0.033	0.699
WC	BMD (30 < age ≤ 45)	6	9471 (GEFO)	0.130 (0.107)	0.844	0.543	0.157 (0.146)	0.282	0.134 (0.204)	0.534	0.176 (0.295)	0.578	-0.003	0.019	0.875
WC	BMD (45 < age < 60)	6	18735 (GEFO)	-0.112 (0.231)	0.629	0.447	-0.147 (0.183)	0.424	-0.215 (0.178)	0.351	0.713 (0.791)	0.286	-0.017	0.013	0.244
WC	BMD (age ≥ 60)	6	22435 (GEFO)	0.271 (0.137)	0.050	0.638	0.282 (0.170)	0.097	0.356 (0.203)	0.221	0.169 (0.639)	0.934	0.006	0.093	0.961
NC	BMD (age ≤ 15)	4	11807 (GEFO)	-0.002 (0.040)	0.957	0.459	0.010 (0.048)	0.834	0.018 (0.058)	0.776	-0.067 (0.152)	0.7701	0.015	0.033	0.699
NC	BMD (15 < age ≤ 30)	4	4180 (GEFO)	-0.061 (0.071)	0.39	0.531	-0.026 (0.089)	0.775	0.014 (0.123)	0.918	0.040 (0.256)	0.890	-0.022	0.055	0.722
NC	BMD (30 < age ≤ 45)	4	9471 (GEFO)	-0.041 (0.062)	0.508	0.132	-0.777 (0.058)	0.185	-0.096 (0.071)	0.269	-0.382 (0.058)	0.185	0.076	0.035	0.160
NC	BMD (45 < age < 60)	4	18735 (GEFO)	-0.065 (0.033)	0.046	0.379	-0.057 (0.041	0.159	-0.063 (0.058）	0.355	0.025 (0.125)	0.860	-0.020	0.027	0.529

NC	BMD (age ≥ 60)	4	22435 (GEFO)	0.015 (0.039)	0.696	0.155	-0.018 (0.038)	0.636	-0.019 (0.041)	0.669	-0.159 (0.110)	0.284	0.039	0.023	0.239
WHR	BMD (age ≤ 15)	42	11807 (GEFO)	-0.545 (0.636)	0.391	0.665	-0.509 (1.699)	0.764	-0.531 (0.830)	0.540	-1.009 (0.741)	0.216	0.013	0.011	0.262
WHR	BMD (15 < age ≤ 30)	42	4180 (GEFO)	-0.034 (0.138)	0.807	0.774	0.010 (0.204)	0.962	0.057 (0.313)	0.857	0.131 (0.432)	0.764	-0.005	0.012	0.690
WHR	BMD (30 < age ≤ 45)	42	9471 (GEFO)	-0.232 (0.822)	0.778	0.065	0.240 (0.894)	0.951	0.160 (1.018)	0.879	-0.633 (0.951)	0.527	0.012	0.014	0.409
WHR	BMD (45 < age < 60)	42	18735 (GEFO)	0.267 (0.438）	0.541	0.819	-0.040 (1.642）	0.981	-0.156 (0.675）	0.823	0.161 (0.503）	0.758	0.003	0.008	0.682
WHR	BMD (age ≥ 60)	42	22435 (GEFO)	0.106 (0.546）	0.846	0.114	-0.147 (1.191）	0.902	-0.086 (0.635）	0.896	-0.453 (0.525）	0.416	0.015	0.007	0.071
ICV	BMD (age ≤ 15)	39	11807 (GEFO)	-0.002 (0.006)	0.687	0.106	0.005 (0.007)	0.53	0.008 (0.008)	0.311	0.007 (0.012)	0.529	-0.006	0.006	0.352
ICV	BMD (15 < age ≤ 30)	39	4180 (GEFO)	-0.005 (0.009)	0.591	0.203	-0.006 (0.012)	0.623	0.002 (0.015)	0.880	-0.033 (0.019)	0.098	0.017	0.011	0.111
ICV	BMD (30 < age ≤ 45)	39	9471 (GEFO)	-0.006 (0.006)	0.317	0.353	-0.010 (0.008)	0.233	-0.008 (0.009)	0.393	-0.008 (0.012)	0.500	0.002	0.007	0.814
ICV	BMD (45 < age < 60)	39	18735 (GEFO)	-0.007 (0.006)	0.237	0.001	-0.006 (0.006)	0.316	-0.004 (0.008)	0.64	-0.011 (0.012)	0.33	0.003	0.006	0.631
ICV	BMD (age ≥ 60)	39	22435 (GEFO)	0.006 (0.005）	0.218	0.008	0.012 (0.005）	0.035	0.018 (0.006）	0.003	0.008 (0.010）	0.428	-0.001	0.006	0.804

BMD: bone mineral density; FA: forearm; FN: femoral neck; LS: lumbar spine; eBMD: estimated heel BMD; TB: total body BMD; GEFO: Genetic Factors for Osteoporosis Consortium; MR: Mendelian randomization; SNP: single-nucleotide polymorphisms; IVW: inverse-variance weighted.

Table S4. Two-sample MR analyses of anthropometric traits on the risk of bone fractures and falls.

Exposure	Outcome	SNP (n)	Outcome sample size	IVW			Weighted Methods				MR egger regression				
							Weighted Median		Weighted Mode		MR egger		Intercept		
				OR (95% CI)	*P* value	*P* heterogeneity	OR (95%CI)	*P* value	OR (95%CI)	*P* value	OR (95%CI)	*P* value	Intercept	se	*P* value
HC	Fractures	7	234973 (GEFO)	0.804 (0.735, 0.879)	1.73E-06	0.670	0.767 (0.677, 0.868)	2.76E-05	0.753 (0.631, 0.900)	0.020	0.711 (0.560, 0.903)	0.038	0.009	0.008	0.328
HC	Falls	7	451179 (GEFO)	1.002 (0.992, 1.012)	0.450	0.060	0.996 (0.988, 1.003)	0.890	0.993 (0.984, 1.001)	0.839	0.997 (0.976, 1.018)	0.891	-0.004	0.008	0.680
WC	Fractures	6	234973 (GEFO)	1.469 (1.363, 1.584)	0.795	0.077	1.383 (1.257, 1.521)	0.659	1.366 (1.060, 1.759)	0.854	0.897 (0.713, 1.129)	0.437	0.047	0.040	0.453
WC	Falls	6	451179 (GEFO)	0.957 (0.846, 1.082)	0.481	0.003	0.968 (0.838, 1.118)	0.500	0.985 (0.854, 1.136)	0.218	0.424 (0.107, 1.683)	0.358	0.014	0.003	0.090
NC	Fractures	4	234973 (GEFO)	1.469 (1.363, 1.584)	0.037	0.590	1.383 (1.257, 1.521)	0.116	1.366 (1.060, 1.759)	0.352	0.897 (0.713, 1.129)	0.971	-0.010	0.014	0.568
NC	Falls	4	451179 (GEFO)	1.009 (0.947, 1.074)	0.783	0.001	0.989 (0.951, 1.028)	0.571	0.983 (0.937, 1.031)	0.527	0.939 (0.736, 1.198)	0.663	0.016	0.027	0.608
WHR	Fractures	42	234973 (GEFO)	0.961 (0.926, 0.998)	0.955	0.081	0.964 (0.921, 1.009)	0.875	0.963 (0.900, 1.030)	0.798	1.003 (0.883, 1.139)	0.856	0.024	0.036	0.796
WHR	Falls	42	451179 (GEFO)	1.072 (0.994, 1.122)	0.250	0.127	0.998 (0.987, 1.010)	1.000	0.979 (0.963, 1.006)	0.976	1.010 (0.989,1.016)	0.372	-0.008	0.004	0.099
ICV	Fractures	39	234973 (GEFO)	1.002 (0.992, 1.012)	0.720	0.881	0.996 (0.988, 1.003)	0.271	0.993 (0.984, 1.001)	0.079	0.997 (0.976, 1.018)	0.779	0.003	0.006	0.605
ICV	Falls	39	451179 (GEFO)	1.002 (0.997, 1.007)	0.494	1.44E-6	1.005 (1.000, 1.011)	0.063	1.003 (0.997, 1.009)	0.364	1.005 (0.993, 1.016)	0.428	-0.002	0.003	0.593

BMD: bone mineral density; FA: forearm; FN: femoral neck; LS: lumbar spine; eBMD: estimated heel BMD; GEFO: Genetic Factors for Osteoporosis Consortium; MR: Mendelian randomization; SNP: single-nucleotide polymorphisms; IVW: inverse-variance weighted.
